# Magnetic resonance imaging and tensor-based morphometry in the MPTP non-human primate model of Parkinson’s disease

**DOI:** 10.1371/journal.pone.0180733

**Published:** 2017-07-24

**Authors:** Michel Modo, William R. Crum, Madeline Gerwig, Anthony C. Vernon, Priya Patel, Michael J. Jackson, Sarah Rose, Peter Jenner, Mahmoud M. Iravani

**Affiliations:** 1 Department of Radiology, University of Pittsburgh, Pittsburgh, Pennsylvania, United States of America; 2 Department of Bioengineering, University of Pittsburgh, Pittsburgh, Pennsylvania, United States of America; 3 McGowan Institute for Regenerative Medicine, University of Pittsburgh, Pittsburgh, Pennsylvania, United States of America; 4 Centre for the Neural Basis of Behavior, University of Pittsburgh, Pittsburgh, Pennsylvania, United States of America; 5 Department of Basic and Clinical Neuroscience, King’s College London, London, United Kingdom; 6 Department of Neuroimaging, King’s College London, London, United Kingdom; 7 Department of Neuroscience, University of Pittsburgh, Pittsburgh, Pennsylvania, United States of America; 8 MRC Centre for Neurodevelopmental Disorders, King’s College London, London, United Kingdom; 9 Institute of Pharmaceutical Sciences, King’s College London, London, United Kingdom; 10 Department of Pharmacy, Pharmacology & Postgraduate Medicine, University of Hertfordshire, Hatfield, United Kingdom; Thomas Jefferson University, UNITED STATES

## Abstract

Parkinson’s disease (PD) is the second most common neurodegenerative disorder producing a variety of motor and cognitive deficits with the causes remaining largely unknown. The gradual loss of the nigrostriatal pathway is currently considered the pivotal pathological event. To better understand the progression of PD and improve treatment management, defining the disease on a structural basis and expanding brain analysis to extra-nigral structures is indispensable. The anatomical complexity and the presence of neuromelanin, make the use of non-human primates an essential element in developing putative imaging biomarkers of PD. To this end, ex vivo T_2_-weighted magnetic resonance images were acquired from control and 1-methyl-4 phenyl-1,2,3,6-tetrahydropyridine (MPTP)-treated marmosets. Volume measurements of the caudate, putamen, and substantia nigra indicated significant atrophy and cortical thinning. Tensor-based morphometry provided a more extensive and hypothesis free assessment of widespread changes caused by the toxin insult to the brain, especially highlighting regional cortical atrophy. The results highlight the importance of developing imaging biomarkers of PD in non-human primate models considering their distinct neuroanatomy. It is essential to further develop these biomarkers in vivo to provide non-invasive tools to detect pre-symptomatic PD and to monitor potential disease altering therapeutics.

## Introduction

Parkinson’s disease (PD) is a clinical syndrome with physical signs consisting of resting tremors, bradykinesia, muscle rigidity, a lack of postural reflexes, “freezing” phenomena and a flexed posture [1]. Non-motor symptoms are also common and include depression, sleep disruptions, autonomic dysfunction and in a sub-set of cases, cognitive impairment, which can progress to dementia [2]. The loss of dopaminergic neurons of the substantia nigra pars compacta is thought to be the pivotal neuropathological event that leads to a progressive degeneration of the nigrostriatal pathway [1]. The development of intraneuronal inclusions of the mutated α-synuclein aggregates, so called Lewy Bodies [3], and their transfer to other cells, implicates the spreading of these inclusions to anatomically connected brain regions leading to a progressive neurodegeneration [4, 5]. The fact that PD-associated cognitive symptoms and non-motor symptoms, are not affected by dopamine replacement [6] also implicates involvement of anatomical structures outside the nigrostriatal axis in the disease process [2]. Ideally, *in vivo* non-invasive biomarkers of these neuropathological changes should be developed to afford an unequivocal differential diagnosis of PD from other related pathologies, but also to provide a means to monitor and stage the progression of the disease and to therefore assess putative therapeutic interventions [7–11].

Magnetic resonance imaging (MRI) and its versatility in assessing a variety of tissue characteristics is potentially well suited to provide non-invasive biomarkers for PD [7, 9, 11–13]. Morphometric structural changes, as indicated by T_2_-weighted images, are thought to provide potential non-invasive biomarkers of PD, but there is considerable variability in these measures [14–19]. The difficulty in achieving an unequivocal clinical diagnosis of PD is likely to contribute to the heterogeneity of imaging findings [8], as any potential confounds of dopamine-replacement medication have not been systematically addressed [12, 20]. To overcome this issue, modeling of PD-relevant disease mechanisms in animals can potentially provide a reasonably homogenous cohort of subjects that would afford the identification of key inclusion and exclusion criteria to improve the differential diagnosis of PD [11, 21]. These animal models also allow us to validate imaging biomarkers against neuropathological measures at the same time point, which is generally not possible in human subjects.

In rodent models of PD, direct neurotoxin lesioning of the nigrostriatal pathway results in an acute hyperintense signal [22, 23], very likely associated with inflammation at the site of injection, in addition to volumetric changes in the midbrain and striatum at longer follow-up post-lesioning [24, 25]. In the sub-acute to chronic phase, these regions gradually produce a hypointense signal due to iron deposits that reduce the T2 signal [19, 26]. There is further evidence that damage to the nigrostriatal pathway also leads to extra-nigral atrophy, especially in cortical regions [25, 27], and dysfunction [28]. Nevertheless, a whole brain analysis using tensor-based morphometry (TBM) revealed a distinct spatio-temporal sequence of progression of tissue atrophy, as well as hypertrophy of the ventricular system [27]. Although animal models provide a more consistent phenotype, the method of disease induction can potentially have dramatic effects on the documented neuropathological changes [25, 29].

It is important to note that the basal ganglia, which is affected in PD, is anatomically quite different in rodents, with for instance the caudate and putamen forming a single structure, the striatum. Primates (including humans) and rodents also have a different distribution and quantity of dopaminergic cells (50% in rodents; 70% in primates) in the substantia nigra and dopamine exerts a slightly different function in the striatum [30]. Dopamine afferents are also far more prevalent in the cortex of humans and primates than in rodents [31]. Rodents do not have neuromelanin, which in primates (including humans) provides the characteristic dark (i.e. nigra) tint to the substania nigra [32]. The array of behaviors that can be assayed in primates is also more complex and akin to the human condition, including psychological changes [30]. As marmosets are easy to handle, survive and reproduce well in captivity [33], they provide a favorable non-human primate (NHP) model that can address some of these complex questions [30]. The systemic administration of MPTP is a common method to induce Parkinson-like behaviors that mimic the human condition [34]. For instance, MPTP in marmosets produces the equivalent of bradykinesia, rigidity and atypical posture [35], in conjunction with a loss of dopaminergic cells from the substantia nigra and tyrosine-hydroxylase (TH)-positive fibers from the caudate and putamen [34, 36]. Nevertheless, there is a lack of resting tremors and no Lewy bodies are formed [35]. Although mutant α-synuclein injections can induce cytoplasmic inclusions equivalent to Lewy bodies, the model remains very variable [37] and therefore is not yet suited to evaluate putative biomarkers.

Nevertheless, marmosets provide a unique opportunity to develop imaging biomarkers that are applicable to the human brain [38, 39]. Indeed, MPTP-treated marmosets have been used to assess changes in brain function using functional and pharmacological MRI [40–42] and more recently a case study using DTI [43], as well as voxel-based morphometry [44]. We extend these findings here to report an integrated analysis of putative MRI biomarkers involving T2-based volume changes in controls and MPTP-treated marmosets of both genders. In addition to high tissue contrast, a high spatial resolution is achieved to avoid potential partial volume effects in anatomical structures that are difficult to delineate. To go beyond a hypothesis-driven region-of-interest analysis, tensor-based morphometry was applied to these scans to statistically contrast localized voxel-by-voxel differences [27].

## Methods

### Marmoset model of Parkinson’s disease

All experimental work was carried out in accordance with the Animals (Scientific Procedures) Act 1986 approved by the Kings College London Ethical Review Committee. In particular, the primate experiments reported were subject to and were carried out under the Animals (Scientific Procedures) Act 1986 detailed in Home Office Project Licence (PPL 70/4986) and approved by King’s College London’s Ethical Review Committee. Procedures complied fully with the guidelines and recommendations set out in the Weatherall Report 2006 on the use of non-human primates in research (https://royalsociety.org/policy/publications/2006/weatherall-report/).

Marmoset care and MPTP treatment were carried out as previously described [45]. Adult common marmosets (*Callithrix jacchus)*, weighing between 200 g and 394 g, were employed in this study (Table 1). All marmosets (n = 11: 6 male, 5 female) were kept in home cages with dimensions of height: 166, width: 140 and depth: 90 cm. The animals’ environment was enriched by installation of viewing turret on top of the cages to mimic height as would be the case in a normal habitat (height: 36cm width: 35cm depth: 50cm) and wooden ladders/perches, hammocks, swings, nesting boxes, multiple feeding platforms and saw dusted floors for forage feeding. Animals were housed in home cages in pairs, as approved by the Home Office inspectorate at King’s College London facilities, in a 12h light/dark cycle at an ambient temperature of 25±1°C and were fed once daily with a diet of bananas, oranges and apples and had free access to food pellets (Mini Marex–E; Special diet Services) and drinking water. All animals had *ad libitum* access to food pellets (Mazuri Primate Diet, Special Diets Services LTD., Witham, UK) and water. Fresh fruit was given once each day with a bi-weekly supplement of vitamin D3. Animals were housed under standard conditions (24±2°C, 50% humidity, and a 12 h light-dark cycle; light on 07:00, lights off 19:00). All animals were group housed.

**Table 1:** Animal characteristics.

Marmoset	Group	Gender	Age (days)	Weight (g)	Lesion Age
1	Control	Male	4147	349	N/A
2	Control	Male	1509	433	N/A
3	Control	Female	3204	311	N/A
4	Control	Female	1088	370	N/A
***Mean***			***2487***	***365***	
***Standard Deviation***			***1243***	***44***	
5	MPTP	Male	4231	298	1542
6	MPTP	Male	3789	373	1480
7	MPTP	Male	4216	378	1480
8	MPTP	Male	3818	235	728
9	MPTP	Female	936	394	343
10	MPTP	Female	2981	200	399
11	MPTP	Female	4146	349	1480
***Mean (SD)***			***3445***	***318***	***1064***
***Standard Deviation***			***1100***	***70***	***510***

To compare the effect of 1-methyl-4-phenyl-1,2,3,6-tetrahydropyridine hydrochloride (MPTP) on brain structures, controls (male n = 2; female n = 2, Charles River, UK) and MPTP-lesioned marmosets (male n = 4; female n = 3) were evaluated. Parkinson-like damage was induced by systemic MPTP (Sigma, UK) administration. The neurotoxin was dissolved in 0.9% saline (2.0 mg/kg) and injected subcutaneously once a day for five consecutive days [46]. After treatment, animals were maintained on a liquid diet (Marmoset jelly, Special Diet Services Ltd., UK, and Complan, Complan Foods Ltd., UK) until they were able to maintain body weight and feed unassisted. The ensuing neurodegeneration is considered a model of PD that reproduces many of the hallmarks of the human condition [47]. Due to biocontainment concerns between the animal facility where marmoset were held and the MR imaging facility, no live imaging of NHP was feasible and instead ex vivo head samples were used for MR imaging.

### Motor function assessment

Following MPTP treatment, all animals displayed stable motor in-coordination, hunched posture, rigidity, bradykinesia. To assess MPTP-induced motor activity, animals were placed individually in motor assessment cages (Perspex-fronted aluminium cages, 50 x 60 x 70 cm) equipped with an array of eight photosensors, and locomotor activity was measured as the number of beam interruptions summed in each 30-min interval over a period of 5 h using an analogue-to-digital converter attached to a Windows PC. For the assessment of motor disability, MPTP-treated animals were scored using an observation-based rating scale while motor activity was being recorded. Scores were as follows: 0–2, alertness; 0–2, checking movements; 0–4, posture; 0–3, balance; 0–2, motility; 0–3, reactions to stimuli and 0–2, vocalization. The motor disability score was obtained by addition of individual scores for each parameter at each observation time point. A score of 0 indicates a normal animal and a score of 18 indicates marked motor disability. On observation days, motor disability was scored every 30 min for a period of up to 5 h. It was previously shown that administration of 12.5 mg/kg levodopa plus 12.5 mg/kg carbidopa only increased motor activity and decreased motor disability significantly when there was ~80% loss of tyrosine-immunoreactive neurons and striatal nerve terminals were observed, while partially lesioned nigrostriatal tract (~50%) did not [48]. Therefore, in order to confirm the full lesion of the nigrostriatal tract following MPTP, before brain scanning, MPTP-treated animals were administered orally with a dose 12.5 mg/kg levodopa methyl ester (Sigma) + 12.5 mg/kg Carbidopa (Sigma). No other drugs were administered.

### Perfusion-fixation

Following treatment and behavioral assessment, the marmosets were deeply anaesthetized with sodium pentobarbitone (Sagatal; c. 80 mg/kg i.p.). Once the thoracic cavity was opened, the animals were intracardially perfused with 0.1M phosphate-buffered saline (PBS), pH 7.4 at 4°C followed by phosphate-buffered 4% paraformaldehyde. Following perfusion, heads were post-fixed for a further 7 days in 4% buffered paraformaldehyde (PFA), washed and maintained in 0.1 M PBS for MR imaging and subsequent histopathology.

### Magnetic resonance imaging

#### Hardware and imaging set-up

All magnetic resonance imaging (MRI) was performed on a 7.0 T horizontal small bore magnet (Varian, Palo Alto, CA, USA) with a custom built 39 mm diameter head quadrature RF coil (David Herlihy, Imperial College London) linked to a Linux-based control console running VnmrJ acquisition software (v2.3, Varian, Palo Alto CA, USA). All acquisitions were performed at room temperature (21°C) on post-mortem samples with brains retained within the skull to avoid artifacts due to dissection or motion, as well as to avoid a tissue-air interface, which could affect cortical measurements. Prior to scanning, skulls were immersed in Fluorinert (Sigma) to provide a hydrogen-free background signal. Ex vivo scanning enables long scanning times that result in high spatial resolution images, which reduce potential confounding imaging artifacts, such as partial volume effects or head motion.

#### T_2_-weighted images

For T_2_-weighted images, a multi-echo multi-slice spin-echo pulse sequence (MEMS) was used with the following parameters: TR = 4200 ms; TE = 10 ms; 8 echo train spaced 10 ms; 8 averages; FOV = 45 mm x 45 mm; matrix = 256 x 256; 0.175 mm in plane resolution; 60 slices; 0.5 mm slice thickness; 0.0875 mm^3^ voxel volume; total scan duration 174 minutes. T_2_-weighted images were produced by summing all echo times (10–80 ms) using VNMRJ (S1 & S2 Figs).

#### Regions of interests (ROI) analysis for volume measurements

**Fig 1:**
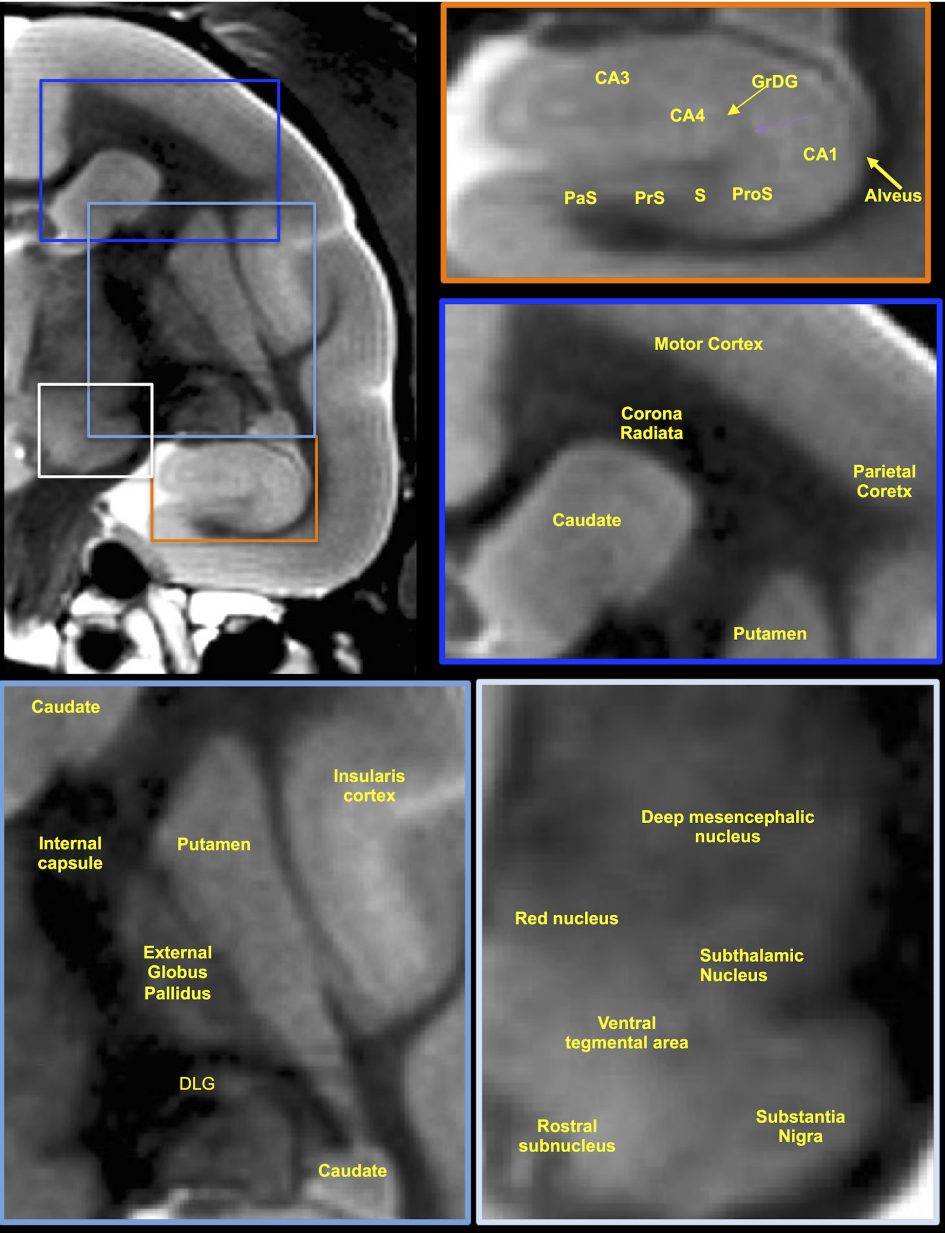
Delineation and distinction of anatomical structures on T_2_-weighted MR images. The high spatial resolution used here (0.0875 mm^3^/voxel) afforded the clear delineation of anatomical regions in the midbrain, such as the deep mesencephalic nucleus, red nucleus, ventral tegmental area, rostral subnucleus. subthalamic nucleus, as well as the substantia nigra. However, the lack of well-defined signal differences between these did not allow separate volumetric quantification of these. The putamen in contrast could be reliable delineated against surround structures, such as the external globus pallidus, the internal capsule, the caudate, as well as the insularis cortex. The caudate was also easily distinguished from surrounding structures, such as the corona radiate, the putamen and overlaying cortical areas, such as the motor and parietal cortex. Although no clear anatomical distinction between motor and parietal cortex was possible based on signal intensity, the distinctive shapes of the corona radiate allowed us to perform separate and consistent measurements within the motor and parietal cortical areas. The hippocampus was clearly resolved on T_2_-weighted images and afforded a reliable measurement with a high inter- and intra-rater reliability (>96%), but sub-structures, such as the CA1-4, granular layer of the dentate gyrus (GrDG), alveus, or the parasubiculum (PaS), prosubiculum (ProS), presubiculum (PrS), subiculum (S) could not be sufficiently and consistently resolved for separate volumetric measurements.

**Fig 2:**
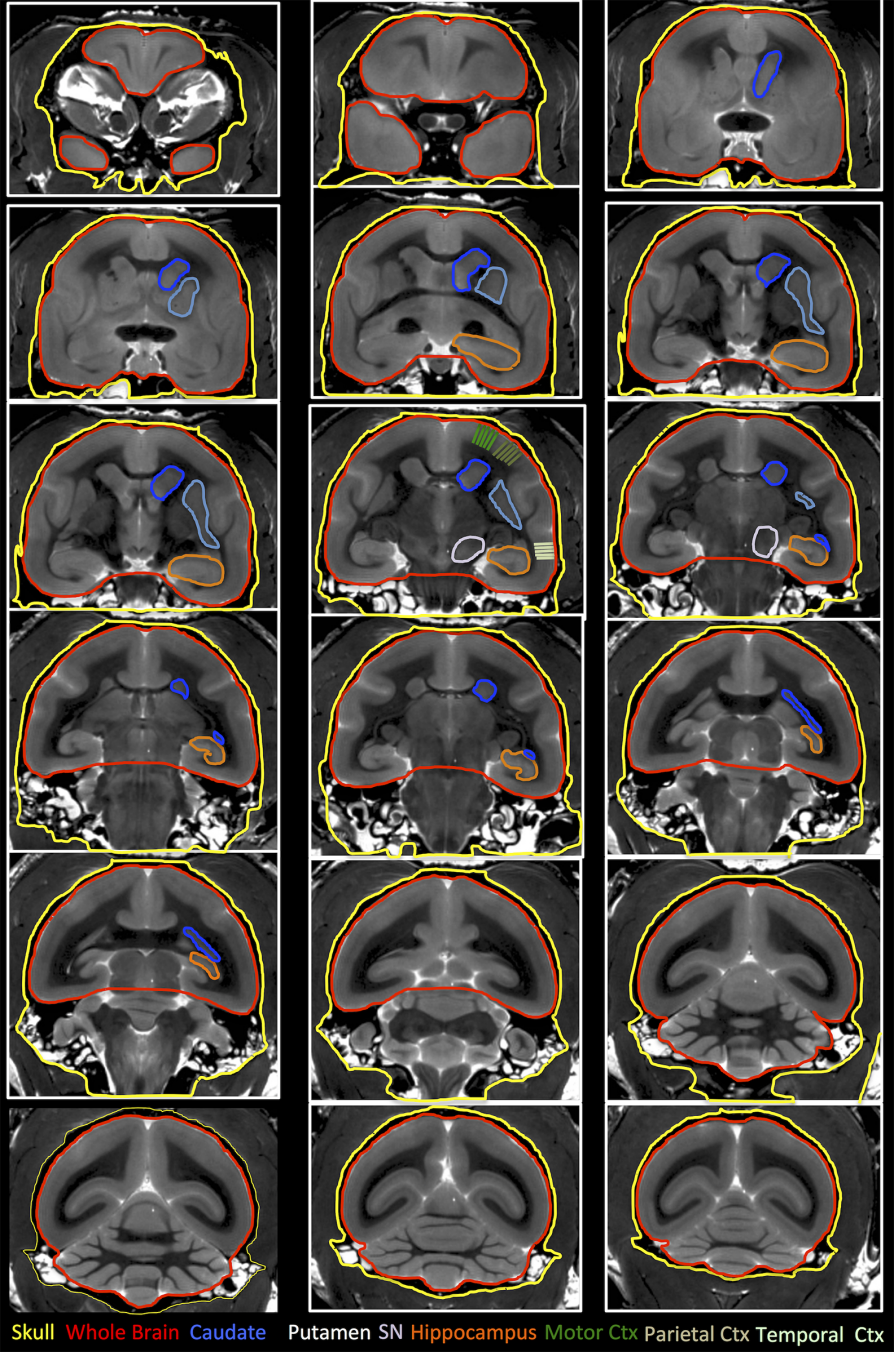
Definition and delineation of regions of interests on T_2_-weighted MRI scans. To control for overall age and growth differences between animals, total skull volume was measured, as this is considered an independent measurement that is not affected by changes in brain tissue and allows a contrast with total brain volume. Using the marmoset atlas [49], the caudate, the putamen, the substantia nigra (SN), and hippocampus were delineated. Cortical thickness was evaluated in the motor, parietal and temporal cortex (Ctx) using five measurements each. For relative signal measurements, a ROI in the visual cortex (purple square) served as internal control.

Manual segmentation was performed on T_2_-weighted MR images (Fig 1) to delineate regions of interest (ROIs) putatively affected in PD, such as the caudate, the putamen, and the substantia nigra. Exquisite image resolution and contrast were achieved allowing precise ROI boundary delineation (Fig 2). Additionally, whole brain volume was measured to account for potential global effects, as well as the hippocampus, which was not likely to be affected by MPTP treatment. Skull volume was also measured to account for difference in animal size and age. As all ROIs were obtained on a per-slice basis, volumes were calculated by multiplying area of ROI by slice thickness (0.5 mm). As cortical regions are difficult to delineate based on signal boundaries, cortical thickness in defined regions was measured as substitute of volumetric change. Regions were defined in accordance with the stereotaxic atlas for the common marmoset [49]. An intra-rater reliability of r = 0.96 (MG) and an inter-rater reliability of r = 0.94 were achieved (MG & PP). Signal measurements were performed within these same ROIs. All manual segmentations were performed using JIM v6.0 software (Xinapse systems, Thorpe Waterville, UK).

#### Tensor-based morphometry (TBM) on T_2_-weighted images

T_2_-weighted structural images were analyzed using a previously reported Tensor-Based Morphometry (TBM) pipeline [50]. In brief, a single control was selected as a canonical reference for population-based 6 degrees-of-freedom (rigid) and 9 degrees-of-freedom (rigid+scaling) registration [51]; the latter allowed us to check for systematic differences in overall brain volume. These registration results were used to generate population mean images for each group. To identify local structural differences between groups, all animals were non-rigidly registered to the control-mean using a high-dimensional “fluid-model” technique [52]. These registration results were used to generate maps of apparent fractional volume-difference (Jacobian determinants) between each animal and the control mean at each voxel in the brain. To identify the location of the most significant differences between groups, we applied t-tests with permutation-based significance testing to each brain-voxel in these volume maps, with multiple comparisons correction using the False Discovery Rate (q<0.05) [53].

### Immunohistochemistry

After MR imaging, brains were removed from the skull and cryo-protected in 20% sucrose solution for 4–6 days. Brain blocks containing the striatum and the ventral mesencephalon were blocked and sectioned coronally in a rostrocaudal orientation, at a thickness of 30 μm at 100 μm intervals throughout the striatum and the ventral mesencephalon using a sliding, freezing microtome. Free-floating striatal and nigral sections were stained for tyrosine hydroxylase immunohistochemistry according to a previously published protocol [46, 48]. Briefly, sections were incubated in a mixture of 2% normal goat serum, 0.05% Triton X-100 (Sigma, St Louis, MO, USA) and the anti-tyrosine hydroxylase (TH) primary antibody raised in rabbit (Pel-Freeze Inc., Rogers, AR, USA) diluted to 1:500 in PBS containing 2% normal goat serum. Incubation was at ambient room temperature for 16 h. After washing in PBS containing 0.05% Triton X-100, the sections were stained with the secondary goat anti-rabbit IgG for 60 min at room temperature and then visualized with avidin–biotin complex (ABC kit, Vectastain; Vector Laboratories, Peterborough, UK) using 3,3,-diaminobezidine as a chromagen. Free-floating sections were mounted onto electrostatically charged slides, and were allowed to dry for 24 h before being dehydrated in increasing grades of ethanol, clearing in Histoclear (BDH) and cover slipping. Sections were observed and photographed using a Zeiss microscope equipped with a Zeiss Axiovision digital camera. Nigral TH-immunoreactive neurons were then counted according to previously published protocol for manual nigral neuronal counting validated against a stereology-based method (optical fractionator) [54].

### Statistics

All graphs and statistical analyses were performed in Prism (v6, GraphPad, San Diego). Data are expressed as mean ± standard deviation. Immunohistochemistry, skull and brain volume comparisons were performed using independent t-tests. All other statistical comparisons were performed using two-way analysis of variance with group (control versus MPTP) as one factor and area of measurements (left/right or ROI) as the 2^nd^ factor. Significant results were followed-up using Sidak post-hoc testing. Post-hoc power analysis were calculated in G*Power (http://www.gpower.hhu.de/en.html) revealing a 1-β>0.8 for all mean comparisons, but insufficient power to perform a correlation analysis. TBM analyses consisted of t-tests (p<0.01) for each voxel pair followed by a correction for multiple comparisons using the FDR (q<0.05).

## Results

### MPTP induced Parkinsonian-like impairments and neuropathology

**Fig 3:**
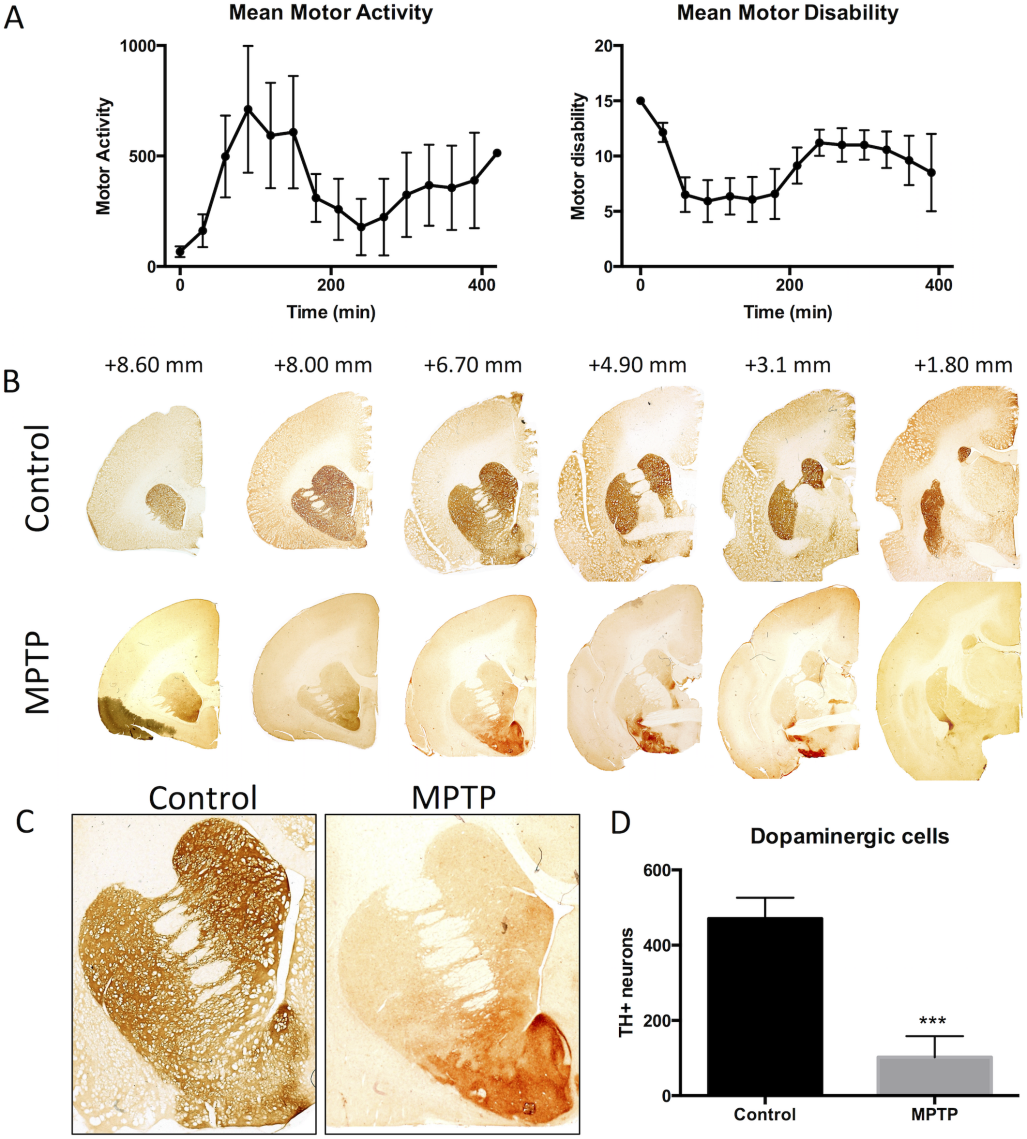
Motor performance and loss of dopaminergic innervation of the nigral-striatal pathway. **A.** Motor activity and motor disability in MPTP treated common marmoset treated with a single dose of 12.5mg/kg levodopa plus 12.5 mg/kg carbidopa. Administration of levodopa led a marked increased in motor activity (left panel) and marked reduction in motor disability (right panel). Both motor activity and motor disability peak at around 90 min of levodopa oral administration. Each data point is a mean ± s.e.m, n = 7. **B.** A denervation of tyrosine hydroxylase (TH) positive dopaminergic fibers is seen in the caudate and putamen after MPTP administration. **C.** A higher magnification of the caudate and putamen further highlights the dramatic loss of dopaminergic fibers in both structures with an even loss throughout the structure. **D.** A quantification of TH+ neurons in the substantia nigra further demonstrated a significant (p<0.001) loss of ~80% of these neurons due to MPTP treatment.

The systemic administration of MPTP to adult marmosets resulted in behavioral impairments, including reduced head-checking movement, akinesia or bradykinesia, hunched posture, reduction of reactions to external stimuli and reduced vocalization. Notably, mean motor activity was 67 ± 24 (mean ± sem), as well as a mean motor disability of 12.1 ± 2.3 at 30 min following acclimatization in the monitoring cages. Administration of levodopa led to a marked and significant increase in motor activity, where at 90 min following levodopa, this was increased more than ten-fold to 711 ± 297 (p<0.01). Motor disability, at this time point, was significantly reduced to 5.9 ± 1.9 (p<0.05). (Fig 3A). Macroscopic images of tyrosine hydroxylase (TH) immunohistochemistry revealed a very significant depletion of dopaminergic innervation of the caudate and the putamen, but also a decrease in dopaminergic neurons in the substantia nigra (Fig 3B). Denervation of dopaminergic fibers was very extensive bilaterally in the caudate and putamen with an extensive decrease in MPTP-treated animals (Fig 3C). In the substantia nigra, a decrease of 73.7% was evident in dopaminergic neurons (p<0.001, Fig 3D). This extensive decrease in the dopaminergic nigro-striatal pathway is a cardinal sign of PD and in conjunction with the behavioral manifestation here verifies the marmoset model used in the current study.

### Manual volumetry detects atrophy in regions associated with Parkinson’s disease

**Fig 4:**
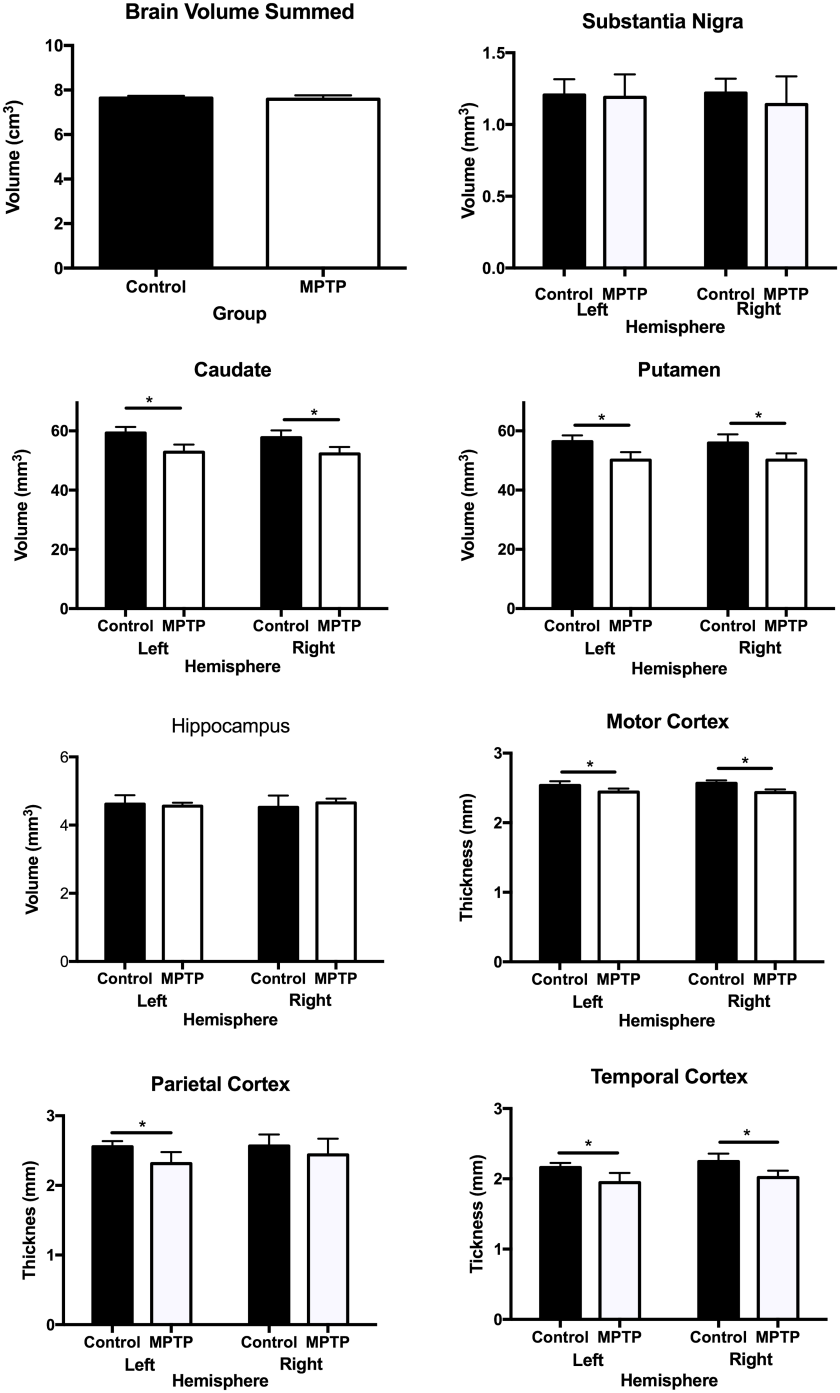
Quantification of anatomical changes due to MPTP treatment. Volumetric analysis (mean+standard deviation) indicated that MPTP treatment did not affect whole brain volume, substantia nigra or hippocampal volume. Nevertheless, a significant (p<0.05) atrophy of was evident in both the caudate (-12%) and the putamen (-11%). There was no laterality effect with both left and right hemispheres showing the same degree of neurodegeneration. There was also a significant effect in motor (p < .05), parietal (p<0.05) and temporal cortex thickness (p<0.05). In the parietal cortex, however, the right hemisphere atrophy did not reach statistical significance.

To assess anatomical changes in MPTP-treated and control marmosets, T_2_-weighted images were used to delineate ROIs that have been implicated in PD. To avoid potential confounds of the size of animals, total skull volume was measured and there was no significant difference between both groups (controls = 11.3 cm^3^; MPTP-treated = 11.15 mm^3^). There was also no significant difference in the total brain volume or the volume of the substantia nigra and hippocampus (Fig 4). However, there was a significant (p<0.05) decrease of 12% in volume in the left and right caudate and a 13% decrease in the putamen. Significant thinning was also observed in the motor cortex (4%), parietal cortex (9%) and temporal cortex (11%) (Fig 4).

### Tensor-based morphometry reveals neuroanatomical changes beyond the nigro-striatal axis

**Fig 5:**
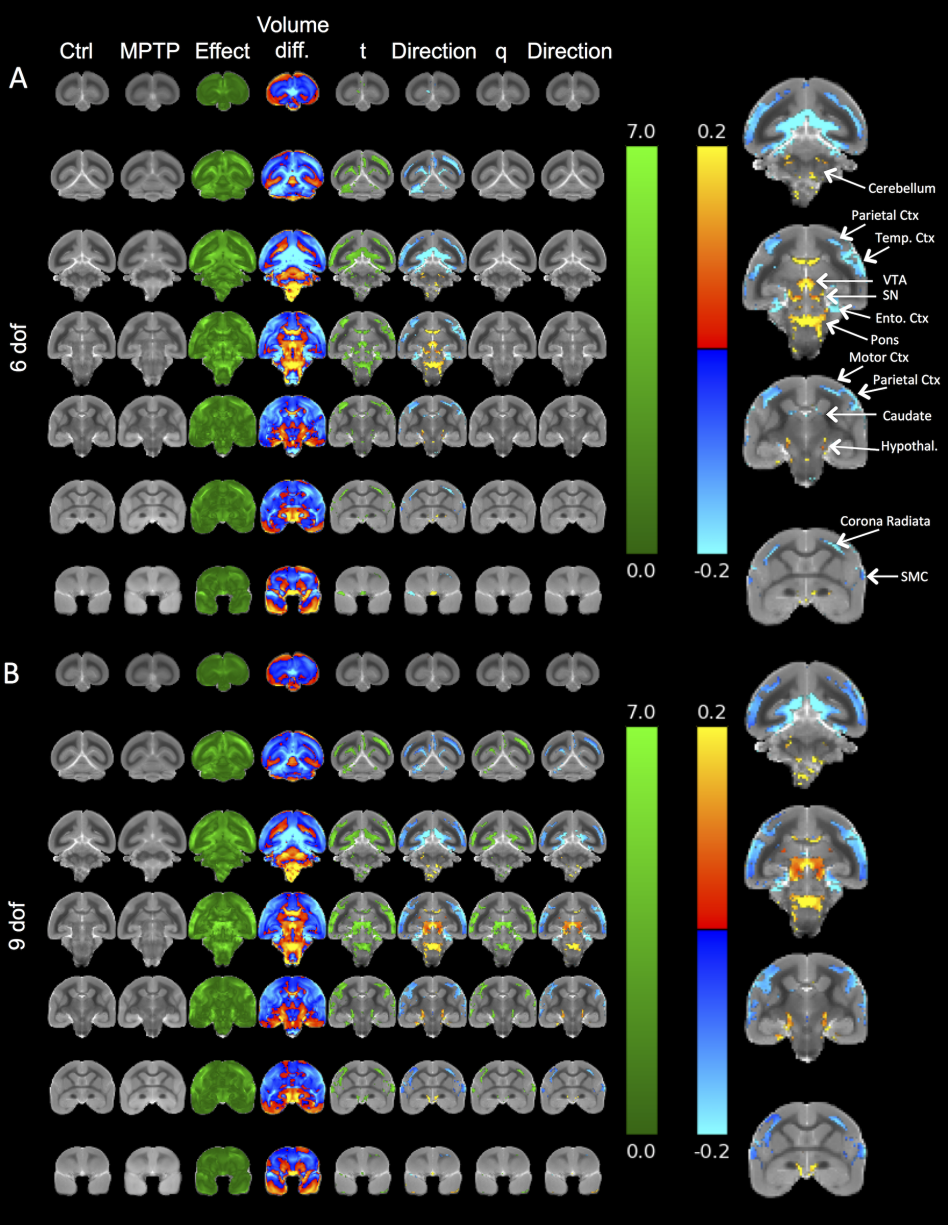
Tensor-based morphometry (TBM) of T_2_-weighted images to contrast anatomical changes in MPTP-treated marmosets. Color scales are for volume difference (green), the raw t-statistical value at each voxel and the direction of the change (warm colors = expansion, cold colors = atrophy). Volume changes which survive multiple comparisons corrections across all voxels in the brain (False Discovery Rate with q<0.05) are also shown. **A.** 6 degrees of freedom (dof) TBM reveals local changes, but does not control for brain size differences. The t maps reveal sub-regional changes in the MPTP-treated animals compared to controls. The directionality map indicates that regional increases are mostly reflected in the ventricular system or the space between the cerebellum and spinal cord. Atrophy is seens mostly in the motor, temporal and parietal cortex, but little change in observed in sub-cortical structures on these comparisons. None of these changes survive a FDR correction for multiple statistical comparisons potentially due to variability and insufficient power. One source of variation that can dramatically affect sub-regional detection of changes is the difference in brain size due to animals of different ages being included here. **B.** Accounting for 9 dof, TBM can reveal sub-region-specific changes that are corrected for global changes (i.e. differences in brain volume). Indeed, this correction produces more consistent effects that survive FDR. Still, cortical areas show a clearer pattern of atrophy compared to sub-cortical regions, with only very subtle effects evident in the caudate or putamen. (Ctx = Cortex, Ento = Entorhinal, Temp = temporal; SMC = somatosensory cortex, SN = substantia nigra, VTA = ventral tegmental area, Hypothal = hypothalamus).

To provide a brain-wide voxel-by-voxel comparison between the control and MPTP-treated groups that does not rely on *a priori* hypotheses as to which regions undergo changes, tensor-based morphometry (TBM) was performed on co-registered and grouped images. TBM analyses were performed using MR images that underwent a 6 degree of freedom (dof) registration, i.e. reflecting regional volume change that might include a difference in whole brain volume (Fig 5A), or a 9 dof registration that accounts for whole brain volume and hence reveals regional changes without being influenced by whole brain atrophy (Fig 5B). The 6 dof TBM indicated volume decreases, especially in cortical areas (parietal, temporal and somatosensory cortex), with a hypertrophy of the ventricular system and the space between the cerebrum and cerebellum. Subtle changes were also evident in the midbrain area, including the substantia nigra, VTA and hypothalamus. Although these differences were statistically significant (p<0.01), they did not survive the more stringent false discovery rate (FDR) multiple comparison correction (q<0.05). By accounting for overall brain size using 9dof registration, the variance between animals was reduced and this increased the power for statistical comparisons and afforded the detection of robust differences (p<0.01) between controls and MPTP-treated animals on a voxel-by-voxel basis after FDR correction (q<0.05). A more defined cortical atrophy was evident, but also a clear atrophy of midbrain structures, such as the substantia nigra and VTA was apparent. On these statistical maps there was no significant volumetric change in the caudate and putamen, despite a 12–13% decrease on volume measurements. This potentially reflects the increased power available to manual ROI delineation, where effects are aggregated across tens or hundreds of voxels compared with the voxel-wise TBM approach.

### Statistical power in small group size NHP experiments is sufficient to establish effect differences, but not correlations

**Fig 6:**
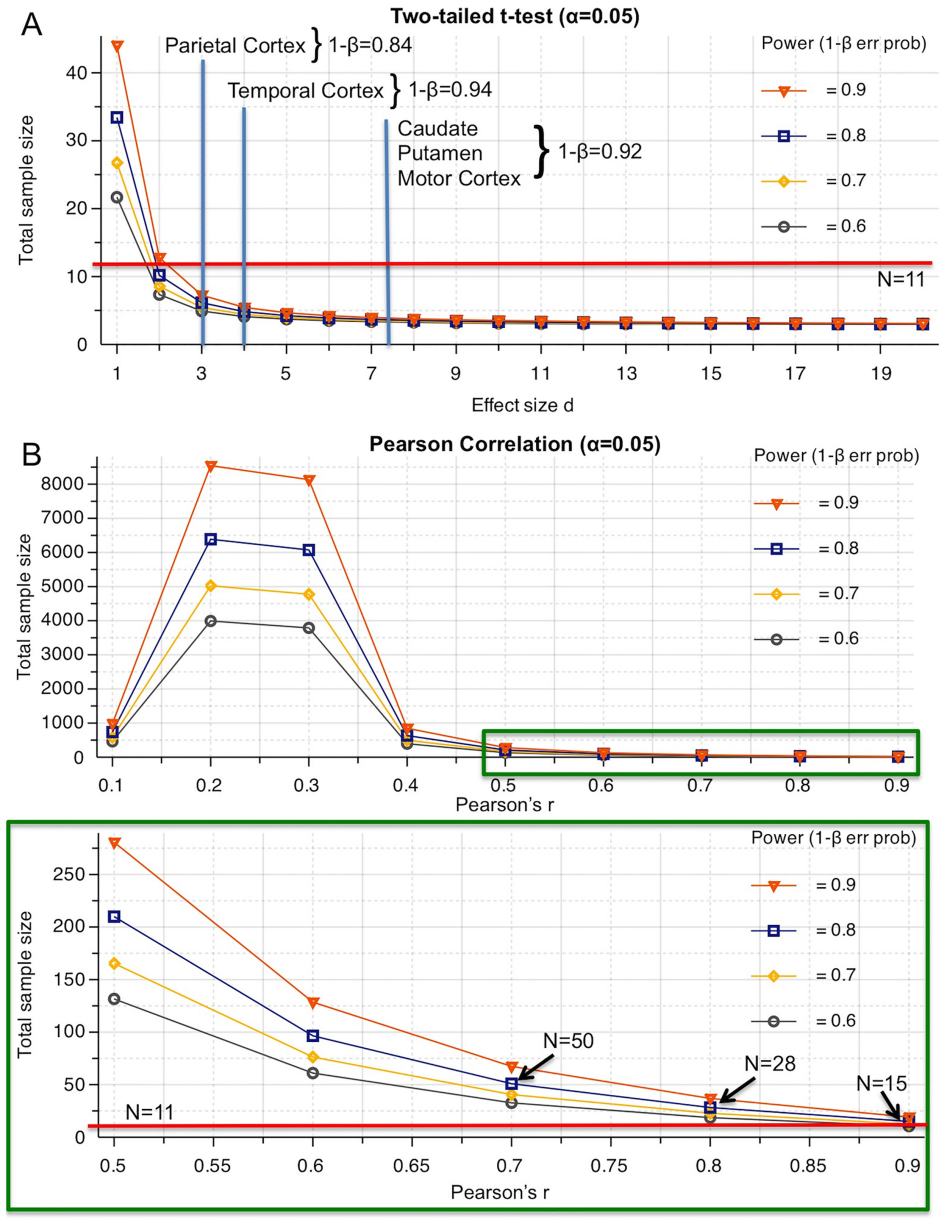
Calculation of statistical power. **A.** Graphical representation of total sample size required for a given effect size to achieve different levels of statistical power for two-tailed (i.e. if no hypothesis regarding direction of effect is available) t-test comparisons. The effect size for different comparisons is indicated in this graph with the corresponding statistical power. With N = 11 in the current experiment, a sufficient statistical power 1-β>0.8 is achieved to avoid Type II errors (i.e. false negatives) with a Type I error (false positives) rate set at 5% (i.e. p<0.05). **B.** Illustration of a power analysis for Pearson’s correlations indicates that small r values (<0.4) require substantial sample sizes to achieve sufficient power to afford a valid comparison at the 5% Type I error rate. In primate studies small sample size are typical due to availability of subjects. A total sample size of 11 is insufficient to achieve an 80% power even with r = 0.9. Significant correlations with medium to high associations of measures would require sample sizes >50 subjects. In rats, we have previously demonstrated that significant correlations between neuropathological measurements and MRI can be performed with N = 15 with high associations (r>0.7) [25, 27].

Only small numbers of non-human primates can typically be enrolled in experimental investigations to address very specific questions. The validity of results from these studies is dependent on Type I (i.e. false positive, acceptable error rate 5%) and Type II errors (i.e. false negatives, acceptable error rate 20%). Establishing volumetric differences here between controls (n = 4) and MPTP-treated marmoset (n = 7) yielded Cohen’s d effects sizes ranging between 3–7.5. For two-tailed t-tests this yields sufficient statistical power to generate valid inferences about group differences, avoiding Type I and Type II errors (Fig 6A). However, smaller anatomical effects, such as those that would be expected in the prodromal stage of the condition, would require larger group numbers. Although these small group sizes are sufficient to statistical compare group-level, main effect differences, they are insufficient for meaningful correlational analyses that aim to associate, for instance, changes in one anatomical structure with those in another (Fig 6B). Only very high correlations (r>0.8) are within reach of these experiments, but would still require N = 28 to achieve a statistical power of 0.8. A major challenge in NHP is variability of measurements. If this can be further reduced correlations with smaller number of subjects are within reach.

## Discussion

The development of non-invasive imaging-based biomarkers is essential to further refine the differential diagnosis of PD and to potentially identify prodromal indicators and markers of disease progression [5, 7, 11, 13, 55]. Magnetic Resonance Imaging (MRI) can indicate changes in anatomy based on volumetric changes (T_2_-weighted images). We here demonstrate that the combination of these types of information can indeed provide biomarkers that in a non-human primate model of PD allow a distinction of control and diseased subjects.

### Regional atrophy in animal models and patients with Parkinson’s disease

In patients with advanced PD, significant regional atrophy is evident with substantia nigra volume loss preceding changes in the basal ganglia and forebrain [5, 55, 56]. In rat models, similar changes are reported [22, 25, 27], but so far little evidence of these changes has been obtained in non-human primate models [42, 57]. We here observed significant regional atrophy in the caudate and putamen using volumetric measures in the MPTP marmoset model, but no clear atrophy was evident in the substantia nigra using hand-drawn ROIs. Nevertheless, using a voxel-by-voxel analysis more subtle structural differences in difficult to delineate areas, such as the substantia nigra, were apparent and consistent with a previous report in the MPTP marmoset model of PD [44]. It is important to note though that signal intensity changes can also affect voxel-by-voxel measures and hence the changes observed here might not necessarily be indicative of a structural change, but a signal change in T2 [58]. A decrease in the T2 signal in the substantia nigra has also been associated with PD using a voxel-by-voxel analysis [15], with R2 and R2* measurements stipulated as putative imaging biomarker [11, 19]. In animals, there is some evidence of hyperintense signals, but this has generally been in cases of direct stereotactic injections of toxins [57, 59] and might reflect an early inflammatory response to injection damage, as a delayed shift to a hypointensve signal has been reported [25, 27, 60]. The use of non-human primates will be essential to disentangle subcortical changes in models of PD, as they capture more accurately the anatomical complexity (caudate and putamen), but also the presence of neuromelanin, which affects the T2 signal of the substantia nigra [42]

Although subcortical changes have been associated predominantly with the motor impairments of the disease, there is also a correlation with the cognitive deterioration in patients [61]. However, to afford a distinction of patients with or without cognitive symptoms, hippocampal atrophy and ventricular hypertrophy [15], as well as the overlying entorhinal cortex [62], have been suggested as key discriminators. Measuring cortical thinning in different areas therefore will potentially be essential to account for different cognitive components of the disease [63]. These anatomical measures will be essential to distinguish PD with or without dementia, but also to address the question if PD with dementia is a natural progression from the milder PD without dementia condition [5, 64]. Current attempts at the diagnostic staging of PD indicate that grey matter changes occur late in the disease state with microstructural changes in WM of nigrostriatal pathway being one of the earliest signs [62], although others argue that this is a reflection of underpowered studies [5]. Disentangling network changes reflective of a progressive condition is consequentially a key component of an early detection and disease staging [11].

### Validity of animal models to develop biomarkers for differential diagnosis and staging of Parkinson’s disease

Nevertheless, defining diagnostic criteria is complicated by poorly defined clinical criteria that allow ambiguity in defining Parkinson’s and related conditions [8, 55]. It is hence difficult to clearly define diagnostic criteria in a clinical population, as different studies might have different inclusion and exclusion criteria to classify patients into different groups [8]. Conversely, this ambiguity in defining different clinical subgroups also affects our ability to clearly define pathological markers in animal models that would replicate the clinical condition [11, 21]. However, in animals specific aspects can be modeled and we can determine the imaging correlates of these pathological changes. For instance, specific damage to the nigrostriatal pathway can be very reliably produced and it is possible to investigate how this will affect structural and signal changes in the animal brain [27, 28, 59, 65]. This provides a neurobiological understanding of the underlying mechanisms of changes observed using diagnostic imaging tools.

Nevertheless, a major shortcoming of these models is the lack of face validity in replicating the disease conditions and the associated progressive degeneration observed in patients [34]. For instance, MPTP in mice or non-human primates is really a model of MPTP damage in humans, i.e. it produces an acute degeneration of dopaminergic neurons that resembles some features observed in PD [30, 32]. To improve the face validity for PD, our models need to improve by, for instance, utilizing local injections of α-synuclein that can spread to different regions and more faithfully replicate the neurobiological conditions of PD [37]. Non-human primate models are essential for imaging biomarkers, due to the anatomical complexity, but also, for instance, the presence of neuromelanin in the substantia nigra that dramatically affects signal intensities [66]. To develop and validate imaging biomarkers useful for a differential diagnosis, models replicating the conditions associated with PSP, MSA, corticobasal syndrome and Richardson’s disease need to be compared [9, 13]. Although ex vivo imaging, as demonstrated here, can be useful to explore biomarkers and define effect sizes and variability for potential in vivo imaging, serial in vivo imaging is absolutely required to determine the usefulness of a putative marker. The use of ex vivo studies to determine feasibility is hence advocated for biomarker exploration, whereas validation of imaging changes will require serial in vivo studies.

Small size NHP studies can reliable detect reasonably small differences between two groups [67]. We here demonstrate that a total sample size of 11 was sufficient to contrast volumetric changes in the sub-chronic MPTP-treated marmosets relative to the controls after chronic lesion maturation (>340 days). However, subtle differences that would distinguish prodromal from control conditions will require larger sample sizes that might be difficult to accomplish for NHP studies. Conducting in vivo repeated measures studies, however, would increase statistical power with the number of repeat measures and hence reduce the total number of subject required to detect subtle difference in brain structure. Still, if the aim is to provide markers for a differential diagnosis using different disease conditions, this will lead to a larger number of group comparison that will decrease statistical power overall, hence requiring again an increase in the number of subjects in each condition. Statistical power is also low for correlation analysis in NHP studies with only very large effect sizes (r>0.8) being within the reach of a reasonable sample size (N = 28). A longitudinal design with a regression analysis also improves statistical power to detect associations between different measurements. Ex vivo studies, as detailed here, are hence be useful to establish large effect sizes to provide measurements that can be used to design and power in vivo longitudinal studies designed to more specifically validate and contrast biomarkers that would be useful in the differential diagnosis of PD [68].

## Conclusions

The development of imaging biomarkers to provide an initial early diagnosis of PD, while affording a differential diagnosis from related Parkinsonian syndromes, will be essential to develop novel therapeutic approaches that aim at altering the disease course [11, 13, 55]. We here demonstrate the utility of T2-based MR imaging for this purpose in a marmoset model of PD. Specifically, a decrease in caudate and putamen volume in conjunction with cortical thinning were observed after MPTP treatment. Voxel-by-voxel comparisons provided evidence of widespread changes in these measures that highlight the extensive anatomical effects that ensue damage to the nigro-striatal pathway. A multi-parametric approach is advised as a key advance to provide a differential diagnosis based on more definitive and direct measures associated with different Parkinsonian syndromes [11]. Animal models can play a major role in the development of these biomarkers, but a high face validity of the models is required to drive these advances and provide neurobiological validations of in vivo imaging changes.

## Supporting information

S1 Fig Animated anterior-posterior T_2_-weighted images of a MPTP-treated marmoset. (GIF)

S2 Fig Serial anterior-posterior T_2_-weighted images of a MPTP-treated marmoset. (TIFF)
