# Supplementary figures and images for: Magnetic resonance imaging and tensor-based morphometry in the MPTP non-human primate model of Parkinson’s disease
Source: PLoS One. 2017 Jul 24;12(7):e0180733. doi: 10.1371/journal.pone.0180733 (PMC5524324; doi:10.1371/journal.pone.0180733)

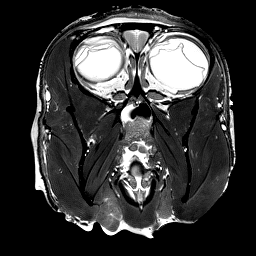

Supplement: S1 Fig — (GIF) [file pone.0180733.s001.gif]

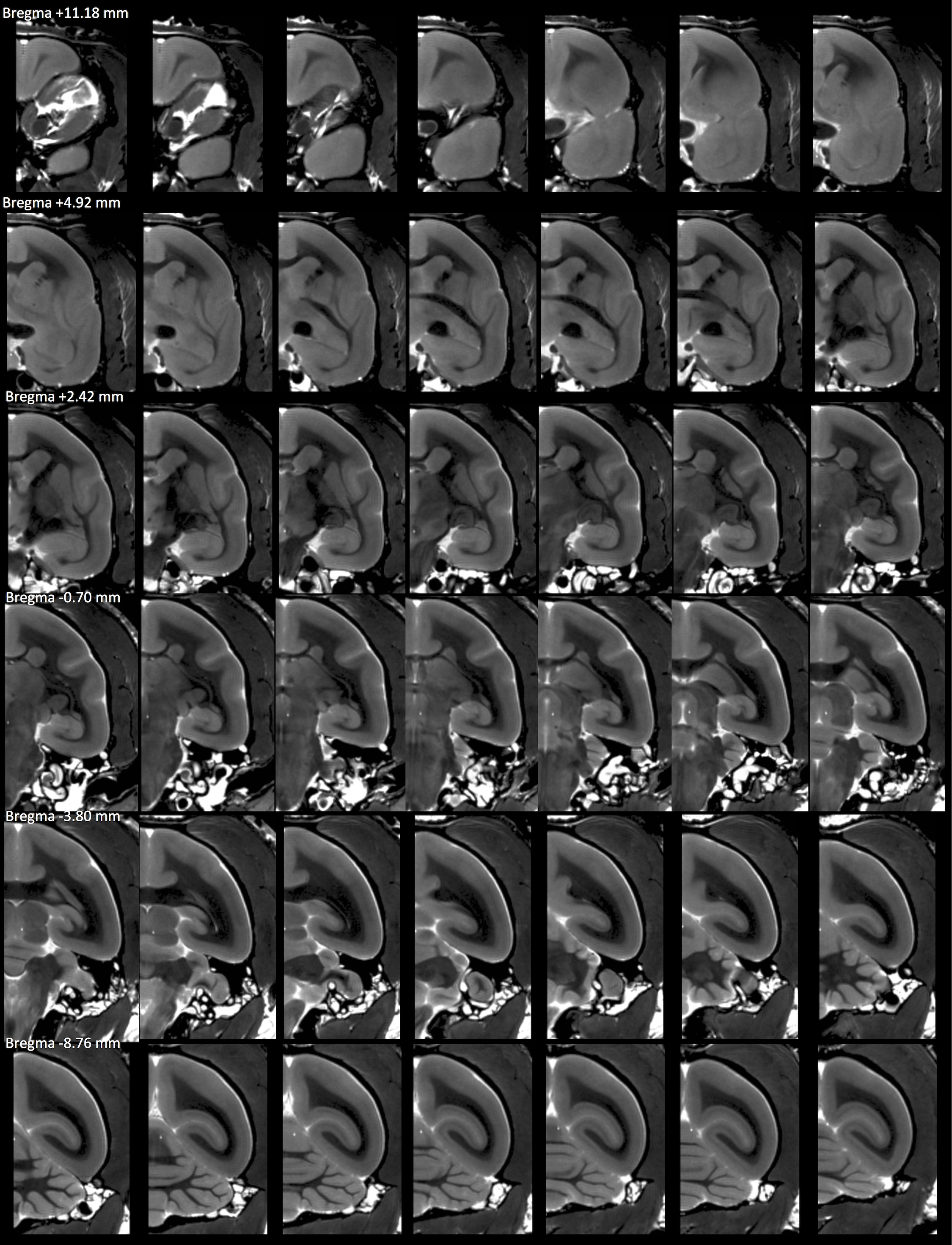

Supplement: S2 Fig — (TIFF) [file pone.0180733.s002.tiff]
